# Where do ICU trainees really look? An eye-tracking analysis of gaze patterns during central venous catheter insertion

**DOI:** 10.1177/11297298241258628

**Published:** 2024-06-10

**Authors:** Philipp K Buehler, Pedro David Wendel-Garcia, Mattia Müller, Marc T Schmidt, Reto A Schuepbach, Quentin Lohmeyer, Daniel A Hofmaenner

**Affiliations:** 1 Institute of Intensive Care Medicine, University Hospital Zurich, Zurich, Switzerland; 2 Center of Intensive Care Medicine, Cantonal Hospital Winterthur, Winterthur, Switzerland; 3 Department of Mechanical and Process Engineering, ETH Zurich, Zurich, Switzerland

**Keywords:** Eye-tracking technology, intensive care units, central venous catheters, cognition, patient safety

## Abstract

**Background:**

There is limited knowledge about gaze patterns of intensive care unit (ICU) trainee doctors during the insertion of a central venous catheter (CVC). The primary objective of this study was to examine visual patterns exhibited by ICU trainee doctors during CVC insertion. Additionally, the study investigated whether differences in gaze patterns could be identified between more and less experienced trainee doctors.

**Methods:**

In a real-life, prospective observational study conducted at the interdisciplinary ICU at the University Hospital Zurich, Switzerland, ICU trainee doctors underwent eye-tracking during CVC insertion in a real ICU patient. Using mixed-effects model analyses, the primary outcomes were dwell time, first fixation duration, revisits, fixation count, and average fixation time on different areas of interest (AOI). Secondary outcomes were above eye-tracking outcome measures stratified according to experience level of participants.

**Results:**

Eighteen participants were included, of whom 10 were inexperienced and eight more experienced. Dwell time was highest for CVC preparation table (*p* = 0.02), jugular vein on ultrasound image (*p* < 0.001) and cervical puncture location (*p* < 0.001). Concerning experience, dwell time and revisits on jugular vein on ultrasound image (*p* = 0.02 and *p* = 0.04, respectively) and cervical puncture location (*p* = 0.004 and *p* = 0.01, respectively) were decreased in more experienced ICU trainees.

**Conclusions:**

Various AOIs have distinct significance for ICU trainee doctors during CVC insertion. Experienced participants exhibited different gaze behavior, requiring less attention for preparation and handling tasks, emphasizing the importance of hand-eye coordination.

## Introduction

The fast-paced milieu in intensive care units (ICUs) presents challenges to healthcare professionals in critical care, with a focus on prioritizing patient safety.^1^ Critically ill patients manifest susceptibility to iatrogenic injuries, owing to the severity of their underlying condition or the inherent risk associated with interventions.^2^ Consequently, the performance of potentially hazardous medical procedures requires meticulous planning and should be carried out by trained practitioners. An example of such an intervention is insertion of a central venous catheter (CVC). Albeit routinely performed in critical care, potential complications such as bleeding, infection, puncture of the artery instead of the vein, pneumothorax, or soft tissue lesions may occur.^3^ Studies estimated a complication rate of 1.5%–15% during CVC insertion.^3^ In light of these considerations, the American Society of Anesthesiologists (ASA) advocates for the implementation of recommendations during CVC insertion.^4^ These include the utilization of standard equipment sets, guidance by real-time ultrasound and adherence to standardized periprocedural processes.^4^ However, there is currently no universally accepted methodology for evaluating the procedure of CVC insertions, especially when performed by new staff or ICU trainees. Traditional methods based on observations by supervisors or experts might be susceptible to potential errors such as observation bias,^5^ and they lack the opportunity to assess situational awareness and visual behavior of the performers, which both have not been characterized in detail during real life CVC insertions. The eye-tracking technology enables precise, objective characterizations of gaze patterns exhibited by professionals, even within critical care settings.^6–11^ It might prove helpful to address the current existing knowledge gaps with regard to CVC insertions. The primary objective of this study was to examine visual patterns exhibited by ICU trainee doctors during CVC insertion using eye-tracking. Additionally, the study investigated whether variations in gaze patterns could be identified between more and less experienced trainees.

## Methods

### Ethics

The competent ethics committee (Local Ethics Committee of the Canton of Zurich, Switzerland, BASEC ID 2017-00798) approved the study protocol. All participating physicians and patients (or their legal representatives in case of incapacity of judgment) gave written informed consent. The study was conducted in accordance with the Declaration of Helsinki.

### Study design and participants

This eye-tracking study was conducted at the interdisciplinary ICU of the University Hospital Zurich, Switzerland. The study was a prospective, real-life investigation, providing a realistic scenario in standard ICU patient treatment rooms. The study team recruited participants from the pool of adult ICU trainee doctors employed at the hospital. To ensure uniformity among participants and to focus on ICU trainees, those with board certification in critical care were excluded. The median of professional experience level was chosen by the study team to be around 6 years of professional experience after university graduation (when doctors get close to board certification and specialist competency), which, in our institution, reflects the transition to more senior functions including supervision of younger fellows. Participants with professional experience below the median experience were considered less experienced, while the others were considered more experienced.

Exclusion criteria were visual disturbances such as monocular vision, achromatopsia, and lack of stereoscopic vision. To minimize biases, data collection was restricted to daytime. The study included patients from diverse medical and surgical fields.

### Study scenario and task

Prior to the experiment, participants were required to complete a baseline questionnaire that assessed demographics, work experience, and past encounters with CVC insertions. Subsequently, all participants were assessed by means of eye-tracking during the insertion of a jugular CVC in a real ICU patient, following our standardized in-house protocol. All CVCs were inserted using ultrasound guidance. All patients were monitored continuously by standard monitoring equipment (electrocardiogram, arterial catheters for invasive blood pressure measurements, and oxygen saturation finger-clips). Participants were instructed to behave as they would in their everyday professional life. The experiment started in an ICU patient room, where the eye-tracking glasses were set-up and a three-point calibration of the eye-tracking device was performed. Eye-tracking recordings were initiated when the participant put the sterile drapes on the patient and started with the CVC insertion. The initial preparation of the CVC on a sterile table and the flushing of the CVC lumen with saline was not subject to analysis. Recordings ended when the participant completed CVC insertion and removed the sterile drapes from the patient. Upon completing CVC insertion, a follow-up questionnaire was administered assessing subjective stress during the experiment, periprocedural workload (Borg scale), and disturbance by the eye-tracking glasses. Moreover, participants were requested to estimate their temporal distribution of visual fixations on various areas of interest (AOI). Potential adverse events and patient complications were documented.

### Outcome measures

**Figure 1.**
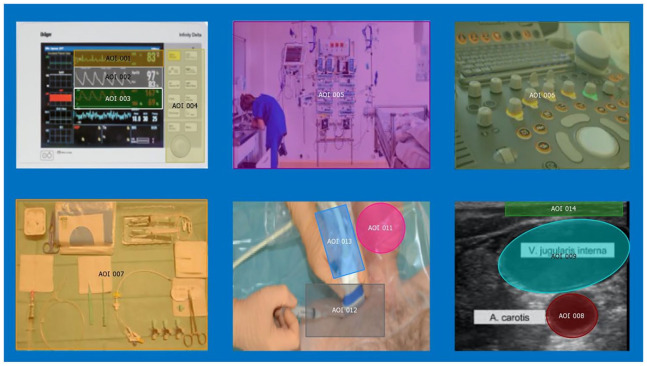
Reference map showing all areas of interest (AOI). AOI 1 = heart rate, AOI 2 = oxygen saturation, AOI 3 = invasive blood pressure, AOI 4 = monitor control panel, AOI 5 = surrounding equipment, AOI 6 = ultrasound control panel, AOI 7 = CVC preparation table, AOI 8 = carotid artery on ultrasound image, AOI 9 = jugular vein on ultrasound image, AOI 10 = white space (irrelevant, not shown), AOI 11 = patient’s neck, AOI 12 = cervical puncture location, AOI 13 = ultrasound probe, AOI 14 = skin insertion site on ultrasound image.

Gaze patterns during CVC insertion were analyzed. Fourteen AOIs considered relevant during CVC insertion were defined by the study team a priori (Figure 1). Primary outcomes were dwell time (cumulated time spent on an AOI, including fixations, blinks, and saccades), first fixation duration (duration of the first fixation of a particular AOI), revisits (frequency of revisiting a particular AOI after looking at other AOIs), fixation count (cumulated number of gaze fixations on a particular AOI), and average fixation time on AOIs. Secondary outcomes were above outcome measures stratified according to professional experience.

### Eye-tracking technology and data recordings

The study was conducted using SMI Eye-tracking Glasses 2 Wireless system (SensoMotoric Instruments, Teltow, Germany; Supplemental Figure 1). In case a participant was myopic/hyperopic, corrective glasses by the manufacturer were available. Gaze tracking was executed at a sampling rate of 60 Hz. Over all distances, the angle of view was measured with accuracy of 0.5°. Resolution of the video recording was 960 × 720 pixels at 30 fps. Using SMI BeGaze Version 3.6 (SensoMotoric Instruments), eye-tracking raw data were processed. The SMI algorithm for fixation analysis was used and all eye-tracking videos were stored in a safe study computer. For data analysis, “BeGaze” Version 3.6 (SensoMotoric Instruments) was used. All visual fixations of participants during CVC insertion were manually assigned to the areas of interest (AOIs), which were mapped in a snapshot reference image.

### Statistical analysis

Descriptive statistics are presented as mean ± standard deviation or median (25%–75% inter-quartile range) depending on data distribution. Data were modeled by linear mixed-effects models, considering eye-tracking measurements as dependent variables, and the specific area of interest as fixed effect while accounting for subject random effect. An additional model considering the interaction between the area of interest and the experience of the participants as fixed effects while accounting for subject random effect was also investigated. *p*-Values for individual fixed effects were obtained by Satterthwaite’s degrees of freedom method. Statistical analysis was performed via a fully scripted data management pathway using the R Environment for Statistical Computing Version 4.3.1. A two-sided *p* < 0.05 was considered to indicate significance.

## Results

Eighteen participating ICU trainee doctors were included. Ten were inexperienced and eight more experienced trainees, respectively. Throughout the study, there were no adverse events. Every participant successfully inserted the CVC without any patient complications. In Table 1, baseline characteristics and results from the pre-experiment questionnaire of the participants are demonstrated. Eight participants (44.4%) were female, the median age was 33.5 years (Interquartile range IQR 31–35), and the median professional experience was 7.5 years (IQR 6–8). Median experience in CVC insertion was rated 4 (IQR 3–5) on a scale ranging from 0 to 5. Table 1 illustrates the number of prior CVC insertions, subjective workload, and two most significant subjective aspects during CVC placement (as subjectively rated). The most important aspects rated were sonographic compression of the vein to distinguish it from artery (30.3%), sonographic control of Seldinger wire position within the vein (24.2%), and safe puncture while aspirating the needle (24.2%).

**Table 1.** Baseline characteristics and results from pre-experiment questionnaire from 18 participants.

Age (years)	33.5 (31–35)
Male gender (%)	10 (55.6%)
Professional experience (years)	7.5 (6–8)
Vision correction	
None	10 (55.6%)
Lenses	3 (16.7%)
Glasses	5 (27.8%)
Experience in placing CVC (scale 0–5)*	4 (3–5)
CVCs inserted before (*n*)	
0–10	2 (11.1%)
11–50	3 (16.7%)
51–100	6 (33.3%)
>100	7 (38.9%)
Importance of vital parameter monitor during CVC insertion (scale 0–5)*	4 (4–5)
Workload before CVC placement (Borg scale 6–20)*	11 (11–12)
Two most important aspects during CVC insertion (%)*	
Patient does not move	5 (15.2%)
Safe puncture while aspirating the needle	8 (24.2%)
Sonographic compression of vein to distinguish from artery	10 (30.3%)
Sonographic control of wire position within the vein	8 (24.2%)
Avoidance of heart rhythm disorders	2 (6%)

CVC: central venous catheter.

Data are presented as numbers (percentages) or median (interquartile range), as appropriate.

* marks a subjective and self-assessed characteristic on a scale.

**Figure 2.**
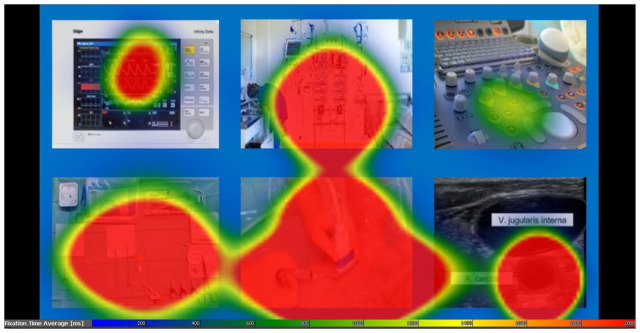
Integrated heat map for dwell time of all participants. More and longer visual fixations lead to a more intense color. Since dwell time was significantly longer on the jugular vein in comparison to the jugular artery, the heat map fails to accurately differentiate between the two.

In Figure 2, a heat map illustrates the dwell time distribution across AOIs by all participants. Not all AOIs were hit by all participants. None of the participants gazed at monitor control panel (AOI 4) and skin insertion site on ultrasound image (AOI 14). Only one participant gazed at the carotid artery on ultrasound (AOI 8).

**Figure 3.**
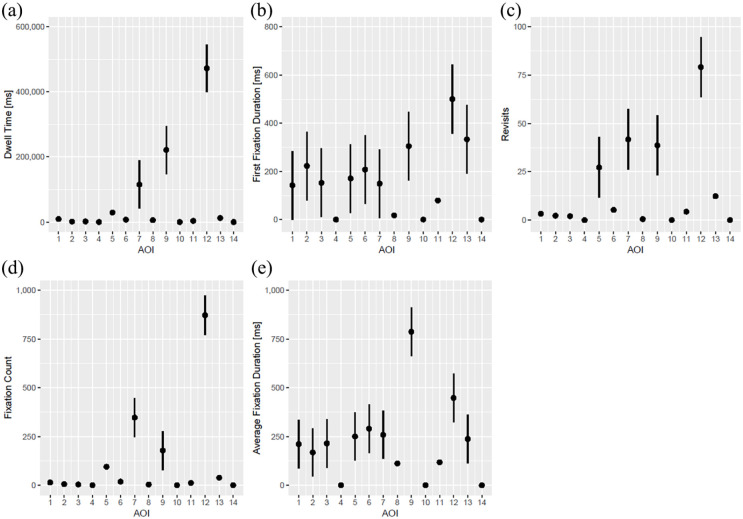
Dwell time (a), first fixation duration (b), revisits (c), fixation count (d), and average fixation time (e) on all AOIs overall.

Figure 3 shows the primary outcome eye-tracking measures overall for all participants, irrespective of their professional experience. Dwell time was significantly higher for CVC preparation table (AOI 7, *p* = 0.02), jugular vein on ultrasound image (AOI 9, *p* < 0.001), and cervical puncture location (AOI 12, *p <* 0.001; Figure 3). First fixation duration was higher for oxygen saturation (AOI 2, *p* = 0.03), ultrasound control panel (AOI 6, *p* = 0.04) jugular vein on ultrasound image (AOI 9, *p* = 0.003), cervical puncture location (AOI 12, *p* < 0.001), and ultrasound probe (AOI 13, *p* < .001). Participants showed more revisits on surrounding equipment (AOI 5, *p* = 0.006), CVC preparation table (AOI 7, *p* < 0.001), jugular vein on ultrasound image (AOI 9, *p* < 0.001), and cervical puncture location (AOI 12, *p* < 0.001), while fixation count was highest for CVC preparation table (AOI 7, *p* < 0.001), jugular vein on ultrasound image (AOI 9, *p* = 0.009), and for cervical puncture location (AOI 12, *p* < .001). Average fixation duration was highest for jugular vein on ultrasound image (AOI 9, *p* < .001) and cervical puncture location (AOI 12, *p* < 0.001; Figure 3). All estimates and all *p*-values corresponding to Figure 3 are presented in Supplemental Table 1.

**Figure 4.**
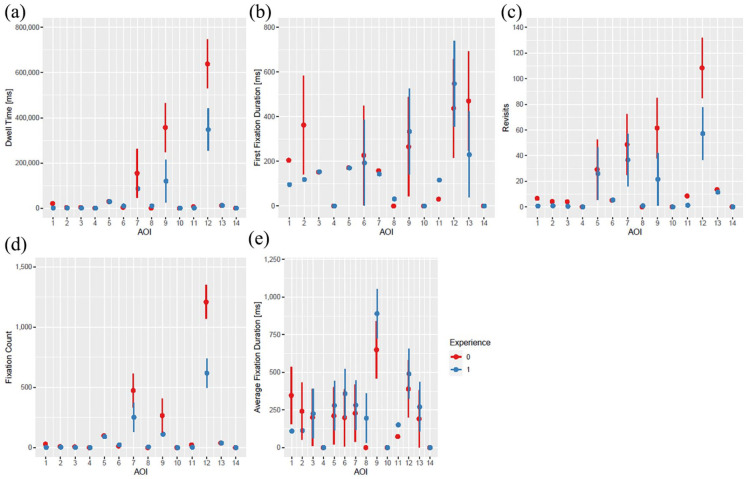
Dwell time (a), first fixation duration (b), revisits (c), fixation count (d), and average fixation time (e) on all AOIs according to experience. The number of “0” (red color) indicates less experience, the number of “1” (blue color) indicates more experience.

Figure 4 illustrates the differences in outcome measures based on the level of experience. It becomes evident that there are key distinctions between the gaze behaviors of more and less experienced participants. Dwell time was shorter in more experienced ICU trainees for jugular vein on ultrasound image (AOI 9, *p* = 0.02) and cervical puncture location (AOI 12, *p* = 0.004). First fixation duration did not differ between more and less experienced participants. Similar to dwell time, revisit numbers on jugular vein on ultrasound image (AOI 9, *p* = 0.04) and cervical puncture location (AOI 12, *p* = 0.01) were decreased in more experienced doctors. Fixation count was smaller in experienced participants only for cervical puncture location (AOI 12, *p* < 0.001), while for average fixation duration no differences could be found between groups (Figure 4). All estimates and all *p*-values corresponding to Figure 4 are presented in Supplemental Table 2.

Table 2 shows results from the post-experiment questionnaire. A minority of participants felt disturbed by external stimuli or monitor alarms (*n* = 3, 16.7%). Estimations of their overall fixation duration on the monitor, patient, and ultrasound screen were 5% (IQR 4%–10%), 40% (IQR 20%–58%), and 50% (IQR 30%–59%), respectively. More than 50% of participants declared that they sonographically visualized the needle-tip during puncture (*n* = 10, 55.6%), checked vital parameters on the monitor during placement of the Seldinger wire (*n* = 11, 61.1%), and also verified the Seldinger wire intravascularly after puncture (*n* = 16, 88.9%).

**Table 2.** Results from the post-experiment questionnaire.

Stress during CVC insertion (Borg scale 6–20)*	12 (11–15)
Easiness of vein localization by sonography (scale 0–5)*	5 (3–5)
Vein puncture assurance/safety (scale 0–5)*	4 (3–5)
Disturbed by external stimuli	6 (33.3%)
Disturbed by vital parameter monitor alarms	3 (16.7%)
Estimated cumulative visual duration on monitor (%)*	5 (4–10)
Estimated cumulative visual duration on patient (%)*	40 (20–58)
Estimated cumulative visual duration on ultrasound image (%)*	50 (30–59)
Sonographic visualization of needle tip during puncture*	10 (55.6%)
Checking vital parameter monitor during placement of Seldinger wire*	11 (61.1%)
Sonographic verification of Seldinger wire intravascularly after puncture*	16 (88.9%)

CVC: central venous catheter.

Data are presented as numbers (percentages) or median (interquartile range), as appropriate.

* marks a subjective and self-assessed characteristic on a scale.

## Discussion

This study used eye-tracking technology to evaluate ICU trainee doctors during CVC insertion, and to compare gaze patterns based on experience. The findings highlight that certain AOIs held varying visual significance during CVC insertion. The preparation table, jugular vein on the ultrasound, and cervical puncture location attracted most visual attention. With regard to experience, dwell time and revisits on jugular vein on ultrasound image and cervical puncture location were decreased in more experienced participants. This could be attributed to improved manual dexterity, better hand-eye coordination, and enhanced spatial understanding in the interpretation of ultrasound images.

The insertion of a CVC plays a crucial role in patient management. However, for ICU trainees, mastering this can be challenging, given the complexity of ICU patients and the potential risk of iatrogenic harm. So far, limited knowledge about periprocedural gaze patterns during CVC insertion is available. This study was able to characterize gaze patterns during CVC in high detail, which would not be possible by means of conventional observations. The results suggest that the jugular vein on ultrasound image and cervical puncture location bear the highest visual importance for ICU trainees. Given the fact that manual handling and interaction between the ultrasound probe and puncture needle can be particularly difficult, especially for trainees, the authors consider these findings plausible within the context of their research, although comparable literature is largely lacking.

To date, studies investigating CVC insertions have primarily been confined to simulated settings, with for example the use of gelatine phantoms and hands-on clinical simulation stations,^12–15^ or immersive video modelings,^16^ not allowing direct comparisons to this work’s real-life scenario. The present findings provide valuable insights from a real-life setting which complement the existing understanding of gaze patterns during CVC insertion. In the authors’ opinion, the application of eye-tracking in this context might have the potential to prevent procedural harm by offering a comprehensive analysis of visual behaviors on important AOIs. Of note, it is worth highlighting that, despite the recognized importance of sonographic differentiation between the vein and the carotid artery during CVC insertion (assessed in questionnaires), only one participant directed attention toward the carotid artery. Reasons for this finding might include lack of training, time pressure, or lack of visual clarity on the ultrasound. Only eye-tracking can offer this accuracy and granularity of visual data, which might ultimately lead to improved staff teaching or periprocedural patient safety in the future. Moreover, the data gathered in this study can play a crucial role in continually improving education and training of ICU professionals, regardless of experience. Such data serves as a valuable resource, allowing for a “feedback on feedback” approach, especially in enhancing coordination and evaluating visual patterns. This recursive feedback mechanism might not only aid in identifying areas of improvement, but can also be pivotal in fine-tuning the training process to address specific challenges faced by doctors at different stages of their career.

From the questionnaires, it emerged that participants considered the patient monitor highly important during CVC insertion and a majority did not feel disturbed by monitor alarms. Nevertheless, the results revealed that participants exhibited low levels of visual fixation on the monitor, as evidenced by relatively short dwell times and infrequent revisits. Particularly when monitors are given less attention during interventions, acoustic alarms become of paramount importance. Alternatively, additional personnel might need to be actively engaged during the intervention to ensure vital parameters are closely monitored by a supporting team member. This underscores a potential false sense of security in cognitive perception among participants. It’s important to recognize that, especially during complex interventions such as CVC insertion, the ability to make frequent revisits to ensure the stability of vital parameters, including heart rhythm, might be a critical skill that could become more pronounced as ICU doctors advance in their professional routine. The discrepancies observed between the perceived importance of the monitor and actual visual attention to it highlight the efficacy of eye-tracking to identify such cognitive misalignments.

Furthermore, participants estimated their cumulative visual duration on AOIs relating to the patient (i.e. AOIs 11, 12, and 14) to be around 40% of the overall time. However, the cumulated dwell time on these AOIs was almost 50% higher in the data analysis. One could speculate that, from a technical point of view, the visual fixation on the ultrasound image, the needle, and vital parameter monitor however might be relevantly more important during CVC insertion, as opposed to the patient. The data can provide insights into hand-eye coordination, the ability for ultrasound image interpretation, and overall visual awareness. This, in turn, can offer valuable feedback about an individual trainee’s progress and proficiency. Eye-tracking thus offers a valuable tool to identify mismatches between subjective evaluations by participants and objective gaze measurements. These findings might be relevant during debriefings or analysis of human errors, which occur frequently in the setting of intensive care medicine.^17^

The authors’ analysis of the professional experience levels demonstrated relevant differences between more and less experienced participants. Dwell time and revisits on the jugular vein on ultrasound and the cervical puncture location were significantly decreased in more experienced physicians, while similar results were found for fixation count on cervical puncture location. The heightened visual attention directed toward these AOIs may suggest that novices require additional time to focus on the jugular vein using ultrasound guidance during needle insertion, probably because of less manual training. Furthermore, they appear to scrutinize interactions between the sonography probe and the puncture needle at the cervical puncture site with greater intensity compared to their more experienced counterparts. In particular, revisits have been characterized as surrogates for behavioral patterns related to monitoring and controlling.^18,19^ In the context of this study, the increased frequency of revisits on relevant AOIs among less experienced doctors could thus be considered a potential surrogate of more frequent control blinks, which experienced participants don’t need to perform anymore.

Additionally, the observations from this research would also endorse the theory that increased professional experience might enhance familiarity with manual procedures, requiring less frequent visual attention. Nonetheless, it is worth noting that Chen et al. proposed a contrasting observation, suggesting that novices allocated significantly less time to gazing at the ultrasound screen compared to experts.^12^ From the authors’ perspective, this discrepancy might arise from differing conditions in real-life scenarios as opposed to simulated study settings or further confounders. In line with the findings from this research, Chen et al. discerned that beginners allocated a notably extended duration monitoring the needle and ultrasound probe compared to experts.^12^ Tatsuru et al. found that more experienced operators let their eye fall outside an ultrasound monitor fewer times than less experienced ones, however also in a simulated setting.^20^ Eye-tracking technology holds the potential to provide profound insights into gaze patterns associated with professional experience. This, in turn, could advance the understanding of learning processes and the acquisition of manual skills in various scientific fields. Of note, the technique has been successfully used as an objective analysis tool, with potential applications such as surgical trainings and performance assessment.^21^ In addition, potential future hand-tracking systems might further contribute to the investigation of hands-eyes coordination, as well as the evaluation of the performance of novices and experts.

This study has strengths, including its practical approach, the evaluation of performance in an authentic ICU environment with critically ill patients, and its innovative contributions to eye-tracking research. The use of contemporary technology ensures that the findings are both current and applicable in today’s medical setting. The methods allowed for a granular analysis, which can be insightful for training programs.

This research has also to account for limitations. It was conducted in a single centric design, probably limiting external validity. Second, the participant number was relatively low.

However, even with a relatively low participant number, this research was still able to discern significant differences, emphasizing the distinction between novices and experts.

Third, the setting in which the study took place, although authentic, might not capture all variations of ICU environments across different institutions. Nevertheless, the authors were able to study a homogenous participant cohort because only ICU trainee doctors were included. Fourth, the patients exhibited varying comorbidities and coagulation profiles, potentially resulting in varying challenges during CVC placement. Finally, the eye-tracking technology cannot link gaze patterns with cognition, which might introduce biases to some extent.

## Conclusions

This study underscores the varied visual significance of different AOIs for ICU trainees during CVC insertion. Gaze patterns distinctively varied based on professional experience. This research was able to identify significant differences according to experience levels, accentuating the disparity between novices and experts. The study’s modern approach, combined with an authentic setting, offers an innovative contribution to eye-tracking research, providing valuable insights for medical training.

## Supplemental Material

sj-pdf-1-jva-10.1177_11297298241258628 – Supplemental material for Where do ICU trainees really look? An eye-tracking analysis of gaze patterns during central venous catheter insertion Supplemental material, sj-pdf-1-jva-10.1177_11297298241258628 for Where do ICU trainees really look? An eye-tracking analysis of gaze patterns during central venous catheter insertion by Philipp K Buehler, Pedro David Wendel-Garcia, Mattia Müller, Marc T Schmidt, Reto A Schuepbach, Quentin Lohmeyer and Daniel A Hofmaenner in The Journal of Vascular Access

sj-pdf-2-jva-10.1177_11297298241258628 – Supplemental material for Where do ICU trainees really look? An eye-tracking analysis of gaze patterns during central venous catheter insertion Supplemental material, sj-pdf-2-jva-10.1177_11297298241258628 for Where do ICU trainees really look? An eye-tracking analysis of gaze patterns during central venous catheter insertion by Philipp K Buehler, Pedro David Wendel-Garcia, Mattia Müller, Marc T Schmidt, Reto A Schuepbach, Quentin Lohmeyer and Daniel A Hofmaenner in The Journal of Vascular Access

sj-pdf-3-jva-10.1177_11297298241258628 – Supplemental material for Where do ICU trainees really look? An eye-tracking analysis of gaze patterns during central venous catheter insertion Supplemental material, sj-pdf-3-jva-10.1177_11297298241258628 for Where do ICU trainees really look? An eye-tracking analysis of gaze patterns during central venous catheter insertion by Philipp K Buehler, Pedro David Wendel-Garcia, Mattia Müller, Marc T Schmidt, Reto A Schuepbach, Quentin Lohmeyer and Daniel A Hofmaenner in The Journal of Vascular Access
