# Supplementary material for: Where do ICU trainees really look? An eye-tracking analysis of gaze patterns during central venous catheter insertion
Source: J Vasc Access. 2024 Jun 10;26(3):957–65. doi: 10.1177/11297298241258628 (PMC12117137; doi:10.1177/11297298241258628)
Supplement: sj-pdf-1-jva-10.1177_11297298241258628 – Supplemental material for Where do ICU trainees really look? An eye-tracking analysis of gaze patterns during central venous catheter insertion [file sj-pdf-1-jva-10.1177_11297298241258628.pdf]

Supplemental Table 2a (Dwell time – Interaction Experience)

Predictors	*Estimates*	*CI*	*P*
AOI [1] * Experience	-19626.64	-215059.61 – 175806.34	.84
AOI [2] * Experience	-1631.06	-197064.04 – 193801.91	.98
AOI [3] * Experience	-1884.94	-197317.92 – 193548.04	.98
AOI [5] * Experience	50.84	-195382.14 – 195483.81	>.99
AOI [6] * Experience	6609.05	-188823.92 – 202042.03	.95
AOI [7] * Experience	-68640.54	-264073.52 – 126792.44	.49
AOI [8] * Experience	9951.14	-185481.84 – 205384.11	.92
AOI [9] * Experience	-237323.05	-432756.03 – -41890.08	**.02**
AOI [10] * Experience	-0.00	-195432.98 – 195432.98	>.99
AOI [11] * Experience	-5977.68	-201410.65 – 189455.30	.95
AOI [12] * Experience	-290457.09	-485890.07 – -95024.11	**.004**
AOI [13] * Experience	-19.42	-195452.40 – 195413.56	>.99
AOI [14] * Experience	-0.00	-195432.98 – 195432.98	>.99

AOI; area of interest, CI; confidence interval. AOI 4 was taken as the reference value.

Supplemental Table 2b (First fixation duration– Interaction Experience)

Predictors	*Estimates*	*CI*	*P*
AOI [1] * Experience	-109.44	-521.87 – 302.99	.60
AOI [2] * Experience	-244.13	-656.56 – 168.31	.24
AOI [3] * Experience	1.34	-411.09 – 413.77	.99
AOI [5] * Experience	-1.41	-413.84 – 411.02	.99
AOI [6] * Experience	-33.94	-446.37 – 378.49	.87
AOI [7] * Experience	-14.50	-426.93 – 397.93	.95
AOI [8] * Experience	31.11	-381.32 – 443.54	.88
AOI [9] * Experience	68.45	-343.98 – 480.88	.74
AOI [10] * Experience	-0.00	-412.43 – 412.43	>.99
AOI [11] * Experience	85.74	-326.69 – 498.17	.68
AOI [12] * Experience	110.62	-301.81 – 523.06	.60
AOI [13] * Experience	-239.82	-652.25 – 172.61	.25
AOI [14] * Experience	-0.00	-412.43 – 412.43	>.99

AOI; area of interest, CI; confidence interval. AOI 4 was taken as the reference value.

Supplemental Table 2c (Revisits – Interaction Experience)

Predictors	*Estimates*	*CI*	*P*
AOI [1] * Experience	-5.92	-44.72 – 32.89	.76
AOI [2] * Experience	-3.29	-42.09 – 35.51	.87
AOI [3] * Experience	-3.50	-42.30 – 35.30	.86
AOI [5] * Experience	-3.29	-42.09 – 35.51	.87
AOI [6] * Experience	0.21	-38.59 – 39.01	.99
AOI [7] * Experience	-12.04	-50.84 – 26.76	.54
AOI [8] * Experience	0.88	-37.93 – 39.68	.97
AOI [9] * Experience	-40.00	-78.80 – -1.20	**.04**
AOI [10] * Experience	0.00	-38.80 – 38.80	>.99
AOI [11] * Experience	-7.25	-46.05 – 31.55	.71
AOI [12] * Experience	-51.25	-90.05 – -12.45	**.01**
AOI [13] * Experience	-2.00	-40.80 – 36.80	.92
AOI [14] * Experience	0.00	-38.80 – 38.80	>.99

AOI; area of interest, CI; confidence interval. AOI 4 was taken as the reference value.

Supplemental Table 2d (Fixation count – Interaction Experience)

Predictors	*Estimates*	*CI*	*P*
AOI [1] * Experience	-26.67	-273.95 – 220.61	.83
AOI [2] * Experience	-5.12	-252.41 – 242.16	.97
AOI [3] * Experience	-5.46	-252.74 – 241.82	.97
AOI [5] * Experience	-6.92	-254.20 – 240.36	.96
AOI [6] * Experience	10.00	-237.28 – 257.28	.94
AOI [7] * Experience	-222.96	-470.24 – 24.32	.08
AOI [8] * Experience	6.25	-241.03 – 253.53	.96
AOI [9] * Experience	-154.46	-401.74 – 92.82	.22
AOI [10] * Experience	0.00	-247.28 – 247.28	>.99
AOI [11] * Experience	-19.12	-266.41 – 228.16	.88
AOI [12] * Experience	-591.17	-838.45 – -343.89	**<.001**
AOI [13] * Experience	-0.25	-247.53 – 247.03	.99
AOI [14] * Experience	0.00	-247.28 – 247.28	>.99

AOI; area of interest, CI; confidence interval. AOI 4 was taken as the reference value.

Supplemental Table 2e (Average fixation duration – Interaction Experience)

Predictors	*Estimates*	*CI*	*P*
AOI [1] * Experience	-236.92	-577.32 – 103.49	.17
AOI [2] * Experience	-129.03	-469.44 – 211.37	.46
AOI [3] * Experience	25.81	-314.60 – 366.22	.88
AOI [5] * Experience	68.36	-272.05 – 408.77	.69
AOI [6] * Experience	161.18	-179.23 – 501.59	.35
AOI [7] * Experience	54.38	-286.03 – 394.78	.75
AOI [8] * Experience	194.84	-145.57 – 535.25	.26
AOI [9] * Experience	240.49	-99.92 – 580.90	.17
AOI [10] * Experience	0.00	-340.41 – 340.41	>.99
AOI [11] * Experience	77.76	-262.65 – 418.17	.65
AOI [12] * Experience	100.34	-240.07 – 440.75	.56
AOI [13] * Experience	79.56	-260.85 – 419.97	.65
AOI [14] * Experience	0.00	-340.41 – 340.41	>.99

AOI; area of interest, CI; confidence interval. AOI 4 was taken as the reference value.
