# Supplementary figures and images for: Where do ICU trainees really look? An eye-tracking analysis of gaze patterns during central venous catheter insertion
Source: J Vasc Access. 2024 Jun 10;26(3):957–65. doi: 10.1177/11297298241258628 (PMC12117137; doi:10.1177/11297298241258628)

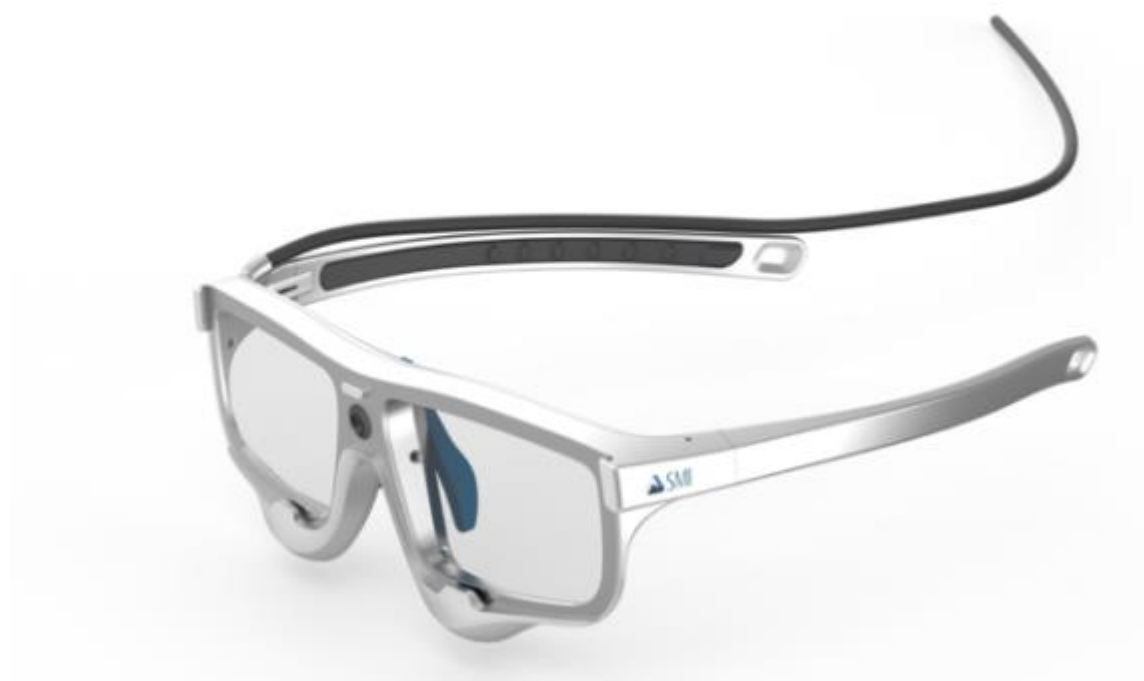

Supplement: sj-pdf-2-jva-10.1177_11297298241258628 – Supplemental material for Where do ICU trainees really look? An eye-tracking analysis of gaze patterns during central venous catheter insertion [file sj-pdf-2-jva-10.1177_11297298241258628.pdf]
