# Supplementary material for: Where do ICU trainees really look? An eye-tracking analysis of gaze patterns during central venous catheter insertion
Source: J Vasc Access. 2024 Jun 10;26(3):957–65. doi: 10.1177/11297298241258628 (PMC12117137; doi:10.1177/11297298241258628)
Supplement: sj-pdf-3-jva-10.1177_11297298241258628 – Supplemental material for Where do ICU trainees really look? An eye-tracking analysis of gaze patterns during central venous catheter insertion [file sj-pdf-3-jva-10.1177_11297298241258628.pdf]

Supplemental Table 1a (Dwell time)

Predictors	*Estimates*	*CI*	*P*
AOI [1]	9208.08	-90654.72 – 109070.88	.86
AOI [2]	1476.86	-98385.93 – 101339.66	.98
AOI [3]	1766.21	-98096.59 – 101629.01	.97
AOI [5]	29266.70	-70596.10 – 129129.50	.56
AOI [6]	7178.64	-92684.16 – 107041.43	.89
AOI [7]	115535.85	15673.05 – 215398.65	**.02**
AOI [8]	5686.36	-94176.43 – 105549.16	.91
AOI [9]	221507.69	121644.89 – 321370.49	**<.001**
AOI [10]	0.00	-99862.80 – 99862.80	>.99
AOI [11]	3468.39	-96394.41 – 103331.18	.95
AOI [12]	472507.89	372645.09 – 572370.68	**<.001**
AOI [13]	12398.89	-87463.91 – 112261.68	.81
AOI [14]	0.00	-99862.80 – 99862.80	>.99

AOI; area of interest, CI; confidence interval. AOI 4 was taken as the reference value.

Supplemental Table 1b (First fixation duration)

Predictors	*Estimates*	*CI*	*P*
AOI [1]	142.26	-57.91 – 342.44	.16
AOI [2]	222.80	22.63 – 422.97	**.03**
AOI [3]	152.90	-47.27 – 353.07	.13
AOI [5]	170.74	-29.43 – 370.92	.09
AOI [6]	207.46	7.28 – 407.63	**.04**
AOI [7]	149.36	-50.81 – 349.54	.14
AOI [8]	17.78	-182.39 – 217.95	.86
AOI [9]	304.70	104.53 – 504.87	**.003**
AOI [10]	0.00	-200.17 – 200.17	>.99
AOI [11]	79.43	-120.74 – 279.60	.44
AOI [12]	500.16	299.99 – 700.34	**<.001**
AOI [13]	333.09	132.92 – 533.27	**<.001**
AOI [14]	0.00	-200.17 – 200.17	<.99

AOI; area of interest, CI; confidence interval. AOI 4 was taken as the reference value.

Supplemental Table 1c (Revisits)

Predictors	*Estimates*	*CI*	*P*
AOI [1]	3.29	-16.15 – 22.72	.74
AOI [2]	2.29	-17.15 – 21.72	.82
AOI [3]	2.00	-17.43 – 21.43	.84
AOI [5]	27.29	7.85 – 46.72	**.006**
AOI [6]	5.29	-14.15 – 24.72	.59
AOI [7]	41.79	22.35 – 61.22	**<.001**
AOI [8]	0.50	-18.93 – 19.93	.96
AOI [9]	38.64	19.21 – 58.08	**<.001**
AOI [10]	0.00	-19.43 – 19.43	>.99
AOI [11]	4.36	-15.08 – 23.79	.66
AOI [12]	79.21	59.78 – 98.65	**<.001**
AOI [13]	12.36	-7.08 – 31.79	.21
AOI [14]	0.00	-19.43 – 19.43	>.99

AOI; area of interest, CI; confidence interval. AOI 4 was taken as the reference value.

Supplemental Table 1d (Fixation count)

Predictors	*Estimates*	*CI*	*P*
AOI [1]	13.93	-119.35 – 147.21	.84
AOI [2]	5.57	-127.71 – 138.85	.93
AOI [3]	4.21	-129.07 – 137.50	.96
AOI [5]	94.71	-38.57 – 228.00	.16
AOI [6]	18.71	-114.57 – 152.00	.78
AOI [7]	346.93	213.65 – 480.21	**<.001**
AOI [8]	3.57	-129.71 – 136.85	.96
AOI [9]	177.57	44.29 – 310.85	**.009**
AOI [10]	0.00	-133.28 – 133.28	>.99
AOI [11]	11.57	-121.71 – 144.85	.86
AOI [12]	871.86	738.57 – 1005.14	**<.001**
AOI [13]	38.36	-94.93 – 171.64	.57
AOI [14]	0.00	-133.28 – 133.28	>.99

AOI; area of interest, CI; confidence interval. AOI 4 was taken as the reference value.

Supplemental Table 1e (Average fixation duration)

Predictors	*Estimates*	*CI*	*P*
AOI [1]	210.99	42.52 – 379.45	**.01**
AOI [2]	168.00	-0.47 – 336.47	.05
AOI [3]	214.56	46.10 – 383.03	**.01**
AOI [5]	250.08	81.61 – 418.54	**.004**
AOI [6]	290.19	121.72 – 458.65	**.001**
AOI [7]	258.82	90.36 – 427.29	**.003**
AOI [8]	111.34	-57.13 – 279.80	.19
AOI [9]	786.92	618.46 – 955.39	**<.001**
AOI [10]	-0.00	-168.47 – 168.47	>.99
AOI [11]	117.40	-51.07 – 285.87	.17
AOI [12]	447.62	279.16 – 616.09	**<.001**
AOI [13]	236.63	68.16 – 405.09	**.006**
AOI [14]	-0.00	-168.47 – 168.47	>.99

AOI; area of interest, CI; confidence interval. AOI 4 was taken as the reference value.
